# Molecular Characterization and Clinical Relevance of Taxonomic Reassignment of Staphylococcus schleiferi Subspecies into Two Separate Species, Staphylococcus schleiferi and Staphylococcus *coagulans*

**DOI:** 10.1128/spectrum.04670-22

**Published:** 2023-02-28

**Authors:** Soe Yu Naing, Birgitta Duim, Els M. Broens, Valentijn Schweitzer, Aldert Zomer, Linda van der Graaf-van Bloois, Coby van der Meer, Luutsen Stellingwerff, Ad C. Fluit, Jaap A. Wagenaar

**Affiliations:** a Department of Biomolecular Health Sciences, Faculty of Veterinary Medicine, Utrecht University, Utrecht, the Netherlands; b Department of Medical Microbiology, University Medical Center Utrecht, Utrecht, the Netherlands; c Certe Medical Microbiology Friesland and Noordoostpolder, Leeuwarden, the Netherlands; University Paris-Saclay, AP-HP Hôpital Antoine Béclère, Service de Microbiologie, Institute for Integrative Biology of the Cell (I2BC), CEA, CNRS

**Keywords:** *Staphylococcus schleiferi*, *Staphylococcus coagulans*, molecular epidemiology, antimicrobial resistance, zoonosis, whole-genome sequencing, medical microbiology

## Abstract

Staphylococcus schleiferi is an opportunistic pathogen in humans and dogs. Recent taxonomic reassignment of its subspecies (*S. schleiferi* subsp. *schleiferi* and *S. schleiferi* subsp. *coagulans*) into two separate species (*S. schleiferi* and *S. coagulans*) lacks supporting data for diagnostic implications and clinical relevance. We aimed to confirm the reclassification of *S. schleiferi* by using genomic and matrix-assisted laser desorption ionization–time of flight (MALDI-TOF) data for a large set of isolates from humans and animals to investigate their molecular epidemiology and clinical relevance. Routine MALDI-TOF analysis and Illumina sequencing were performed on 165 *S. schleiferi* isolates from the Netherlands. With 33 publicly available genomes, the study included 198 genomes from 149 dogs, 34 humans, and 15 other sources. The Type Strain Genome Server was used to identify species in the genomes, and the MALDI-TOF MS database was extended to improve species differentiation. MALDI-TOF did not discriminate between *S. schleiferi* and *S. coagulans.* Genome phylogeny distinguished the two species in two monophyletic clusters. *S. schleiferi* isolates originated from humans, while *S. coagulans* isolates were found in animals and three human isolates clustering with the animal isolates. The sialidase B gene (*nanB*) was a unique marker gene for *S. schleiferi*, whereas the *chrA* gene was exclusive for *S. coagulans*. The *mecA* gene was exclusively detected in *S. coagulans*, as were the *lnu*(A), *blaZ*, *erm*(B/C), *tet*(O/M), and *aac*(6′)-*aph*(2′′) genes. The MALDI-TOF database extension did not improve differentiation between the two species. Even though our whole-genome sequencing–based approach showed clear differentiation between these two species, it remains critical to identify *S. schleiferi* and *S. coagulans* correctly in routine diagnostics.

**IMPORTANCE** This study clearly shows that *S. schleiferi* is a concern in human hospital settings, whereas *S. coagulans* predominantly causes infections in animals. *S. coagulans* is more resistant to antibiotics and can sometimes transmit to humans via exposure to infected dogs. Even though genome-based methods can clearly differentiate the two species, current diagnostic methods used routinely in clinical microbiology laboratories cannot distinguish the two bacterial species.

## INTRODUCTION

Staphylococcus schleiferi is a biosafety level 2 opportunistic pathogen in humans and companion animals. Often recognized as a veterinary pathogen, *S. schleiferi* can cause otitis externa and pyoderma in dogs and cats. In humans, most cases are nosocomial infections, including bacteremia, endocarditis, urinary tract infection, wound and surgical site infection, and medical device-related infection (1, 2). Previous studies suggested that humans could acquire this organism via contact with dogs (1, 3–5). Nevertheless, molecular evidence is still needed to confirm this potential zoonotic transmission. Methicillin resistance has also been reported in both human and dog isolates (6–8), and multidrug-resistant strains are frequently isolated from companion animals (9), which poses a significant public health risk and veterinary concern.

However, epidemiological understanding of human *S. schleiferi* infections is limited, since the real incidence has been underreported due to identification errors (2). Traditionally, *S. schleiferi* had two subspecies classifications based on coagulase activity. *S. schleiferi* subsp. *schleiferi*, a coagulase-negative staphylococcus (CoNS), was first isolated from a human clinical specimen in France in the late 1980s (10). Later, *S. schleiferi* subsp. *coagulans*, a coagulase-positive staphylococcus (CoPS), was isolated from the external ear of a dog in 1990 in Japan (11). Traditional identification methods often misidentified *S. schleiferi* subsp. *coagulans* as Staphylococcus aureus, Staphylococcus pseudintermedius, or other CoPS, whereas further identification might not be performed for *S. schleiferi* subsp. *schleiferi*, because a CoNS might be considered less clinically relevant (2, 4). From a clinical perspective, accurate bacterial identification is critical for case management, because misidentification as S. aureus often results in more aggressive treatments than treatment options for CoNS. Currently, routine diagnostic tests lack the discriminatory power to differentiate between these two subspecies. For example, matrix-assisted laser desorption ionization–time of flight mass spectrometry (MALDI-TOF MS) can detect *S. schleiferi* only on a species level. Previous studies attempted to differentiate subspecies using phenotypic and genotypic characteristics (12, 13). Both studies concluded that they were not distinguishable, highlighting the need to review the subspecies classification. This taxonomic ambiguity continued until Madhaiyan et al. in 2020 used genome-based taxonomy indicators to reclassify both subspecies as two separate species, *S. schleiferi* and *S. coagulans* (14). However, the promotion of subspecies to novel species has remained inconclusive and controversial, since taxonomic reassignment was still based on incomplete 16S rRNA gene sequences and insufficient genome information (15), thus requiring more supporting genomic data to characterize the differences between them.

The new taxonomic reclassification and differences in epidemiology prompted the importance of understanding what differentiates the two species for diagnostics implications and clinical relevance. The main objectives of this study were (i) to investigate if whole-genome sequencing (WGS) using a large set of isolates from humans and animals supports the reclassification, (ii) to study the molecular epidemiology and clinical relevance of both species, and (iii) to apply genomic data to improve identification in routine diagnostics using MALDI-TOF MS.

## RESULTS

### Comparative genomics and molecular epidemiology of *S. schleiferi* and *S. coagulans*.

WGS analysis based on the genome BLAST distance phylogeny (GBDP) method clearly differentiated between *S. schleiferi* (*n* = 32) and *S. coagulans* (*n* = 166) (Table 1). All *S. schleiferi* isolates (32/32) originated from humans, whereas 89.7% (149/166) of *S. coagulans* were isolated from dogs, 1.8% (3/166) from humans, and 8.4% (14/166) from other animal hosts. Human infections with *S. schleiferi* were most commonly bloodstream infections (34.3%; *n* = 12), followed by wound infections (8.6%; *n* = 3). The three *S coagulans* isolates were isolated from humans with a wound infection, otitis externa, or from a nasal swab. Most dogs were infected with *S. coagulans* causing otitis externa (69.1%; *n* = 103), followed by pyoderma (11.4%; *n* = 17). No *S. schleiferi* isolates showed any urease activity, whereas 73.9% of *S. coagulans* (*n* = 122) isolates were positive for urease. The specimen information is summarized in Table 1, and the metadata of each isolate are listed in Table S1 in the supplemental material.

**TABLE 1:** Phenotypic and clinical characteristics of *S. schleiferi* and *S. coagulans* isolates in humans and dogs

Characteristic	All		Humans		Dog	
	%	*n*	%	*n*	%	*n*
Total genomes	100	198	17.7	35/198	75.3	149/198
This study	83.3	165	77.1	27	89.9	134
Public	16.7	33	22.9	8	10.1	15
Genome-based species delineation						
*S. schleiferi*	16.2	32	91.4	32	0	0
*S. coagulans*	83.8	166	8.6	3	89.7	149
Clinical condition						
Otitis externa	53.5	106	2.9	1	69.1	103
Pyoderma	8.6	17	0	0	11.4	17
Bacteremia	6.1	12	34.3	12	0	0
Wound infection	3.5	7	8.6	3	2	3
Brain infection	1	2	5.7	2	0	0
Unknown/other	27.3	54	48.5	17	17.5	26
Urease activity at 48 h^*a*^						
Positive	73.9	122/165	7.4	2/27	88.1	118/134
Negative	26.1	43/165	92.6	25/27	11.9	16/134

a Urease assays results were only available for isolates from the Netherlands that were included in this study (*n* = 165).

**FIG 1:**
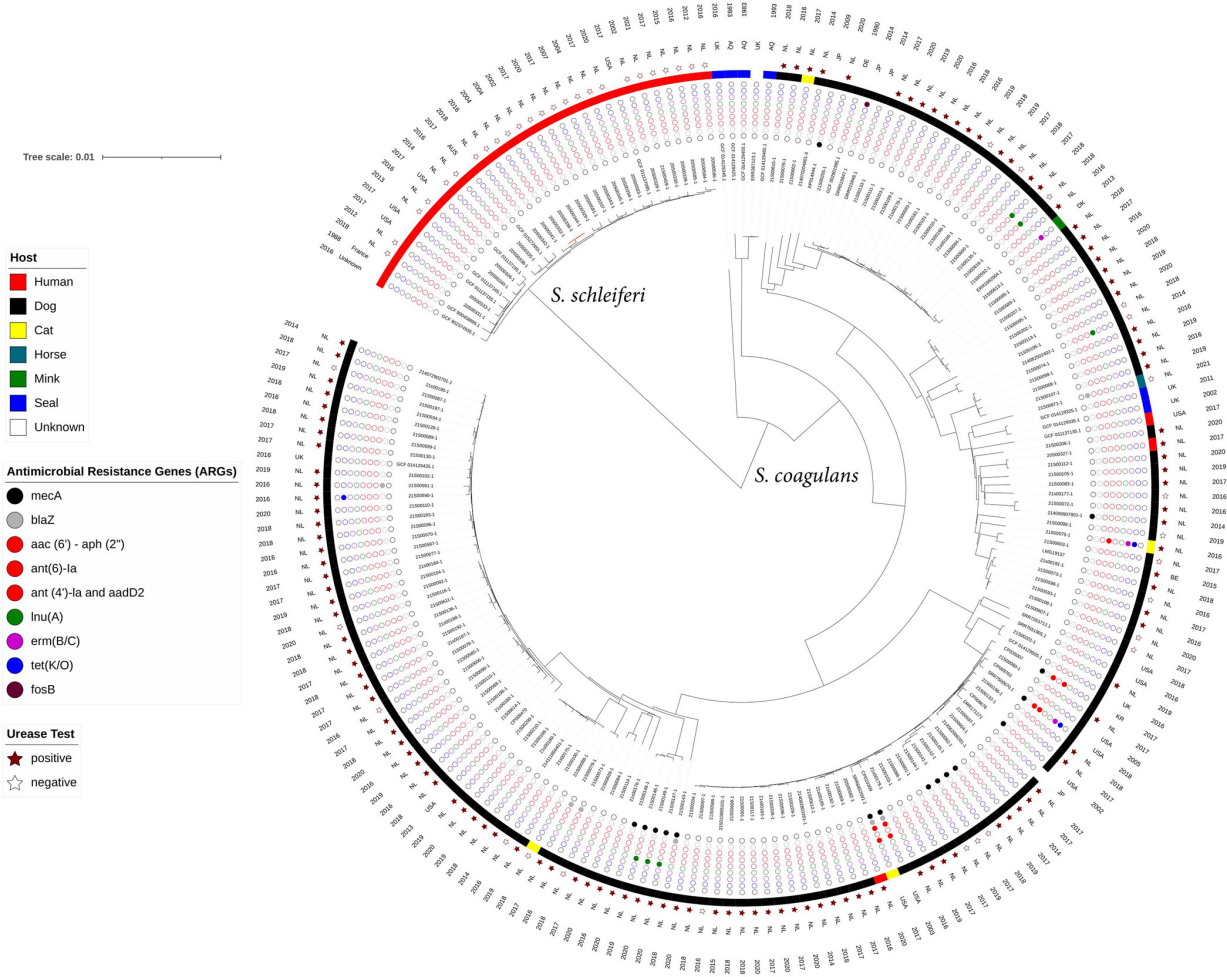
Phylogenetic tree showing the diversity and relatedness of *S. schleiferi* and *S. coagulans* isolates from different hosts, years, and isolation places. AQ, Antarctica; AUS, Australia; BE, Belgium; DE, Germany; DK, Denmark; JP, Japan; KR, Korea; NL, the Netherlands; UK, United Kingdom; USA, United States. The colored squares indicate the host type, and the filled colored circles indicate the presence of ARGs. The filled stars indicate positive urease tests. The absence of any symbol indicates lack of data from public genomes.

The phylogenetic analyses captured the genomic epidemiology of *S. schleiferi* and *S. coagulans*, which formed two monophyletic branches. Both species belonged to distinct clades and had different host ranges (Fig. 1). The phylogenetic tree displayed that *S. schleiferi* isolates were recovered from human samples and closely clustered together in one lineage, whereas *S. coagulans* isolates were found in animals, mostly in dogs, other animals, and three humans. Notably, three Dutch *S. schleiferi* isolates (20S00332-1, 20S00336-1, and 20S00344-1) belonged to the same cluster with 10 to 15 differences in single-nucleotide polymorphisms (SNPs). These isolates were recovered from human patients with a wound infection, central nervous system infection, or a bloodstream infection in the same hospital in the Netherlands between 2002 and 2004. The two Dutch human isolates of *S. coagulans* (20S00327-1 from a wound infection and 20S00582-1 from an ear infection) and one U.S. human isolate from a nasal swab (GCF 0111371135.1) were closely related to dog isolates from the Netherlands.

In terms of antimicrobial resistance, no acquired antimicrobial resistance genes (ARGs) were detected in *S. schleiferi* isolates, whereas 17.4% of *S. coagulans* isolated from dogs (*n* = 26) carried ARGs conferring resistance to beta-lactam (*blaZ*), aminoglycoside [*aac*(6′)-*aph*(2″), *ant*(6)-*la*, *ant*(4′)-*la*, and *aadD2*], lincosamide [*lnu*(*A*)], tetracycline [*tet*(*K/O*)], and erythromycin [*erm*(*B/C*)]. Among them, 15 (10.2%) of *S. coagulans* dog isolates (*n* = 147) and 1 cat isolate were methicillin resistant, carrying the *mecA* gene. All methicillin-resistant *S. coagulans* isolates were phenotypically resistant to oxacillin, and routine quantitative PCR results confirmed the presence of *mecA* genes. There was no phylogenetic clustering corresponding to isolation country, year, or clinical conditions of the isolates. ARGs corresponding to each genome are displayed in Fig. 1 and listed in Table S1.

**FIG 2:**
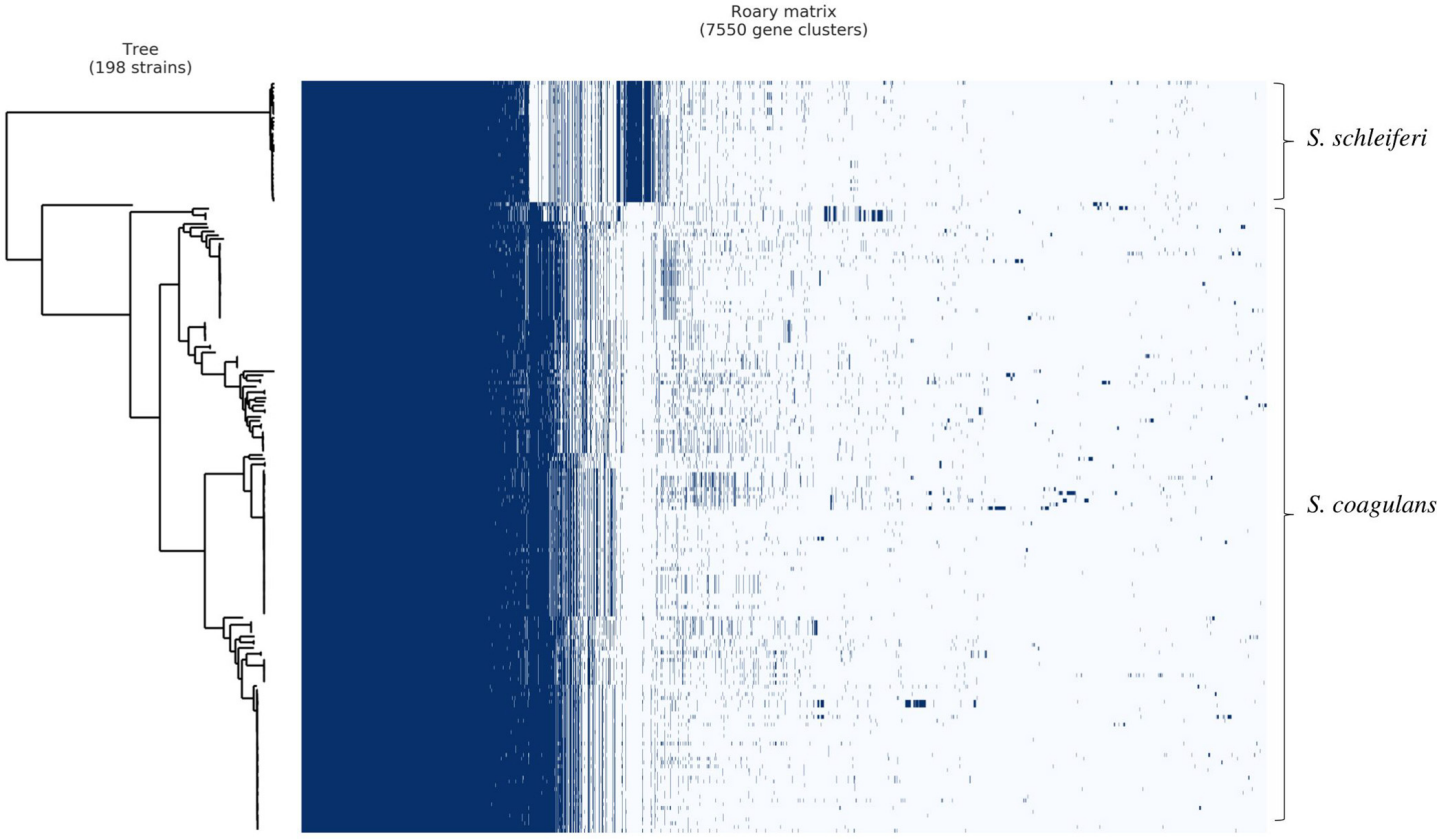
The pan-genome matrix, showing the absence and presence of core and accessory genes in *S. schleiferi* and *S. coagulans* isolates. Dark blue, presence of gene; white, absence of gene.

Comparative genomics analysis showed that *S. schleiferi* and *S. coagulans* isolates shared a total of 1,543 core genes and carried similar virulence factors, such as microbial surface components recognizing adhesive matrix molecules with genes encoding exotoxins, including putative staphylococcal enterotoxin (*SE*), and γ-hemolysins. A genome-wide association study (GWAS) was performed to specify the genes associated with the two species. GWAS analysis identified a set of species-specific genes for both species (Fig. 2; Table S2 and S3). Among them, the sialidase B (*nanB*) gene was exclusively found in *S. schleiferi* isolates, whereas the chromate transport protein (*chrA*) gene was uniquely present in *S. coagulans* isolates except for two seal isolates (*P* ≤ 0.05, Bonferroni correction).

### MALDI-TOF MS database extension to improve differentiation.

**FIG 3:**
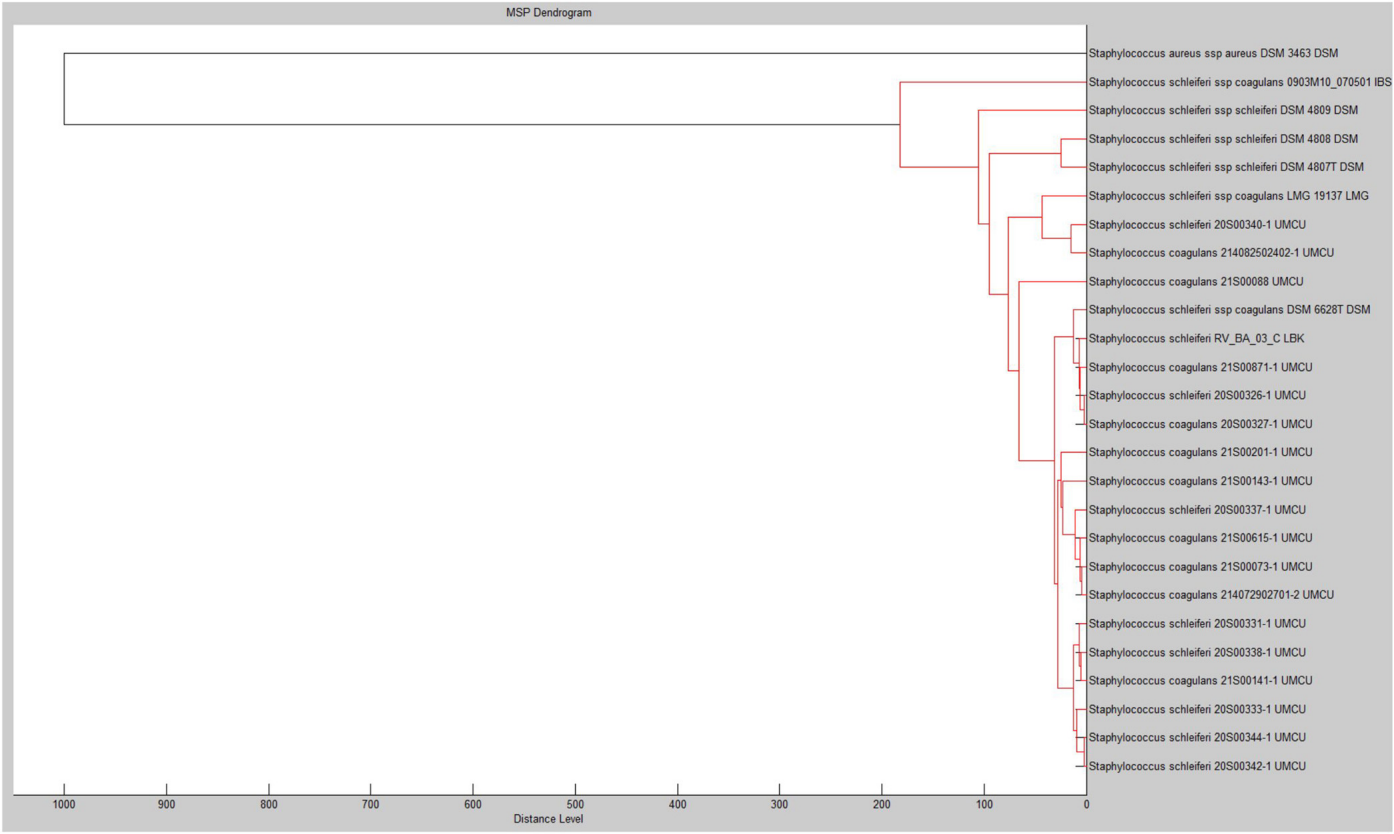
MSP dendrogram of *S. schleiferi* and *S. coagulans* using S. aureus as an outgroup. Distance level indicates the similarity level between isolates, with 0 indicating complete similarity and 1,000 indicating complete dissimilarity.

The cluster analysis of MALDI-TOF MS spectra showed that *S. schleiferi* and *S. coagulans* were closely clustered together, as shown in the main spectrum profiles (MSP) dendrogram in Fig. 3. MALDI-TOF using Bruker database 5627 identified both species as *S. schleiferi* (100%; *n* = 46) in a category A species consistency, with the score value ranging between 2 and 2.29. The extension of the MSP database subsequently resulted in a category B consistency warning for all isolates (*n* = 46), with the score value ranging between 2 and 2.29, indicating the uncertainty about the true identifications of *S. schleiferi* and *S. coagulans*. The database extension failed to differentiate between *S. schleiferi* and *S. coagulans* (Table S5).

## DISCUSSION

This study demonstrated that a genome-based species delineation method provides the high discriminatory power for *S. schleiferi* and *S. coagulans* which cannot be performed by routine identification tools, including MALDI-TOF MS. This is the first study to confirm the species reassignment and elucidate the molecular epidemiology of *S. schleiferi* in human infections, in comparison with *S. coagulans* from both humans and dogs. The addition of publicly available genomes from different hosts and countries also captured a more comprehensive understanding of genomic diversity and host ranges of these pathogens. Our comparative genomic analysis resulted in a set of species-specific biomarkers that can be used to develop an accurate identification scheme to facilitate diagnostics and surveillance. The chromate transport protein (*chrA*) gene was exclusively found in *S. coagulans* isolates. A previous study characterized the *chrA* gene as one of the five MALDI-TOF identification biomarkers to differentiate *S. schleiferi* subsp. *schleiferi* and *S. schleiferi* subsp. *coagulans* (16). In contrast to our findings, those authors identified *chrA* as a unique marker of *S. schleiferi* subsp. *schleiferi*, but at the time of writing their article, the subspecies were not reclassified yet and those authors did not sequence the whole genome of these isolates. Thus, our study showed that species differentiation can be based on the presence of the gene *chrA* to differentiate *S. coagulans* (*chrA*-positive) from *S. schleiferi* (*chrA*-negative) isolates. Previous studies have shown that an extension of the MSP database improves the identification of rare or closely related species in diagnostic laboratories (17). Since WGS showed clear separation of two species, the current study attempted to improve the MALDI-TOF MS identification by extending the MSP database. However, this attempt was unsuccessful, and further work needs to examine if species-specific proteins identified in this study can be used for improving routine diagnostics. In addition, we used bioMérieux Vitek MS to examine if it could identify accurately the two species. However, bioMérieux Vitek MS also did not discriminate between the two species (Table S4).

As MALDI-TOF analyses include the peaks only from ribosomal proteins, future work should examine to capture the complete spectrum of all proteins and determine the differences in peaks according to two species. Previous research also suggested urease activity as one of the phenotypic tests to differentiate between two species (18). In contrast to previous findings, our findings showed the variation of urease activity in *S. coagulans* isolates (Table 1). Despite the fact that urease activity was absent in all *S. schleiferi* isolates, a urease test alone is not sufficient to distinguish the two species. The standard coagulase test is still currently used in some places to differentiate between the two species based on coagulase activity. However, the coagulase test using coagulase rabbit plasma with EDTA is not reliable. This is in agreement with previous research findings in 2016 (12) and from a 1994 study by Vandenesch et al. that demonstrated that both species can promote clotting of rabbit plasma in the standard tube test due to a pseudo-coagulase activity that can give false-positive reactions in the coagulase tube test (19).

In terms of pathogenicity in human infections, *S. schleiferi* was recovered exclusively from human samples and the majority were isolated from bacteremia and wound infections, although it cannot be excluded that there is another unsampled reservoir. Little is known about its epidemiology, and only one study performed extensive investigation of an outbreak associated with surgical site infections in Breda Hospital, the Netherlands, in 1998 (20). That investigation involved environmental sampling, a case-control study, and molecular typing of outbreak-related and environmental strains (20). However, the source of *S. schleiferi* in that hospital outbreak was unknown. In our study, we identified a potential protracted outbreak in the hospital based on similar SNPs between three *S. schleiferi* isolates from the same hospital in the Netherlands between 2002 and 2004.

The genomic characterization revealed that all *S. schleiferi* isolates carried sialidase B (*nanB*), which is a bacterial neuraminidase that promotes the bacteria’s survival in mucosal environments by utilizing host ligands for adherence, biofilm formation, and immunomodulation (21). Thus, the presence of *nanB* suggested that *S. schleiferi* expressed this marker potentially for mucosal colonization and host specificity.

Notably, two Dutch human isolates of *S. coagulans* (from a wound and an ear infection) belonged to the dog cluster from the Netherlands, suggesting that these two human strains of *S. coagulans* were most likely to be transmitted from dogs. These findings are in agreement with previous reports from human infections associated with exposure to dogs colonized with *S. coagulans* (3–5). One limitation in the current study was that there were no available patient demographics, including pet ownership or exposure to confirm a zoonotic event.

In terms of antimicrobial resistance, there was a remarkable difference in resistance patterns between two species. ARGs were detected mainly in *S. coagulans* of dog isolates, while no known resistance genes were discovered in *S. schleiferi* isolates. Drug resistance observed in dog isolates may be explained by the fact that *S. coagulans* strains are exposed to antimicrobials for an extended period given the type of infections commonly seen in dogs (i.e., ear and skin) (22). Among dogs carrying ARGs, methicillin resistance gene (*mecA*) detection was highest, followed by aminoglycoside and lincosamide resistance genes. Further work should investigate the role of mobile genetic elements and plasmid-carried ARGs in both species and different hosts.

To conclude, the current study provides new understanding of the genomic epidemiology of *S. schleiferi* and *S. coagulans* that suggests that *S. schleiferi* is considered a human pathogen associated with nosocomial transmission and infection, whereas *S. coagulans* is an animal pathogen with occasional spillover events in humans. These findings can be useful for infection prevention control for *S. schleiferi* in hospital wards, whereas bacterial sampling of household pets should be initiated in cases of *S. coagulans* infections in humans. The use of a genome-based species delineation method provided the highest discriminatory power and should also be considered an alternative approach for the identification of closely related species, such as *S. schleiferi* and *S. coagulans*. Yet, it remains critical to develop a cost-effective way to differentiate *S. schleiferi* and *S. coagulans* in routine diagnostics, given the importance of accurate identification of these species in clinical practices.

## MATERIALS AND METHODS

### Isolate selection.

A total of 165 animal isolates were collected between 2002 and 2021 from dogs (*n* = 134), cats (*n* = 3), and a horse (*n* = 1) at the Veterinary Microbiological Diagnostic Centre of Utrecht University and from humans (*n* = 27) at the University Medical Centre Utrecht and Certe Medical Microbiology Friesland and Noordoostpolder in the Netherlands. An additional set of isolates (*n* = 33) were selected from public databases (NCBI and the Sequence Read Archive) and included in the genomic analysis. The complete set of genomes (*n* = 198) represented 149 isolates from dogs, 35 from humans, and 14 from other animals. Isolates from the current study were identified as *S. schleiferi* using MALDI TOF-MS with a Bruker Microflex MALDI TOF-MS system, Biotyper software, and MBT-BDAL-5627 MSP library 5267 (Bruker, Germany). *S. schleiferi* isolates with MALDI identification scores of ≥2.0 were included in the study.

### Urease test.

To assess the urease activity of bacterial isolates for differentiation purposes, a few colonies from fresh cultures were inoculated into standard commercial urease tubes (Biotrading, the Netherlands), followed by incubation for 48 h at 37°C. A color change from yellow to pink or purple was considered a urease-positive reaction.

### Whole-genome sequencing.

DNA isolation was performed using the DNeasy UtraClean microbial kit (Qiagen Gmbh, Germany) according to the manufacturer's protocol. The sequencing was performed using Illumina NextSeq sequencing with 1 × 150-bp reads by the Utrecht Sequencing Facility, Utrecht, the Netherlands. The Illumina library was prepared with a final DNA concentration of 2 ng/μL using the Nextera XT library prep kit (Illumina, USA) according to the manufacturer’s protocol.

### Genomic analysis.

All sequences were trimmed with TrimGalore v 0.4.4 (23), assembled using SPAdes v3.14.1 (24), and annotated with Prokka v1.11 (25). The quality of all sequences was checked with Checkm v1.1.3 (26), and only genomes with a contamination threshold of <5% and completeness threshold of >98% were included in the analysis. The comparative genome analysis was performed using Roary v3.13.0 (27) and Scoary v1.6.16 (28). A phylogenetic tree was constructed based on Genome BLAST Distance Phylogeny (GBDP) distances provided by TYGS output (29). Interactive Tree of Life v6.0 (30) was utilized to visualize the metadata of the genomes in a mid-rooted phylogenetic tree. ResFinder v4.0 (31) was used to identify the acquired ARGs.

### Genome-based species identification.

The Type Strain Genome Server (https://tygs.dsmz.de/) was used to differentiate between *S. schleiferi* and *S. coagulans* (29). The species delineation was based on the GBDP method using the digital DNA-DNA hybridization values provided by the TYGS platform (29).

### MALDI-TOF Bruker Database extension.

To improve the differentiation between the two species, main spectrum profiles (MSPs) of *S. schleiferi* (*n* = 8) and *S. coagulans* (*n* = 10) were added to the existing Bruker biotype database 5627 (Table S4). MSPs of these isolates were generated as previously described (17). The extended database was validated using 46 isolates (23 *S. schleiferi* and 23 *S. coagulans* isolates) by performing routine identification in duplicate using the existing Bruker MBT-BDAL-5627 MSP library with and without the new MSPs.

### Ethical statement.

Ethical approval was not required for this study, as the bacterial isolates used in this study were obtained as part of routine diagnostics.

### Data availability.

The sequence data have been deposited in the European Nucleotide Archive (ENA) under study accession number PRJEB53744.
